# AR-induced ZEB1-AS1 represents poor prognosis in cholangiocarcinoma and facilitates tumor stemness, proliferation and invasion through mediating miR-133b/HOXB8

**DOI:** 10.18632/aging.102680

**Published:** 2020-01-24

**Authors:** Xingming Jiang, Jinglin Li, Weina Wang, Zengtao Hu, Canghai Guan, Yuqiao Zhao, Wenzhi Li, Yunfu Cui

**Affiliations:** 1 Department of General Surgery, Second Affiliated Hospital of Harbin Medical University, Harbin 150086, China; 2 Department of Anesthesiology, Second Affiliated Hospital of Harbin Medical University, Harbin 150086, China

**Keywords:** cholangiocarcinoma, prognosis, ZEB1-AS1, miR-133b, HOXB8

## Abstract

Zinc finger E-box binding homeobox 1 antisense 1 (ZEB1-AS1) has displayed vital regulatory function in various tumors. However, the biological function of ZEB1-AS1 in cholangiocarcinoma (CCA) remains unclear. In this study, we confirmed that ZEB1-AS1 expression was increased in CCA tissues and cells, respectively. Upregulated ZEB1-AS1 was related to lymph node invasion, advanced TNM stage and poor survival of CCA patients. ZEB1-AS1 exhibited high sensitivity and specificity to be an independent poor prognostic factor of patients with CCA. Functionally, knocking down ZEB1-AS1 attenuated tumor cell stemness, restrained cellular viability *in vitro* and *in vivo*, and inhibited CCA cell migration and invasion by reversing epithelial-mesenchymal transition. For the mechanism, androgen receptor (AR) directly promoted ZEB1-AS1 expression, and further ZEB1-AS1 increased oncogene homeobox B8 (HOXB8) by sponging miR-133b. In addition, malignant phenotypes of CCA promoted by ZEB1-AS1 dysregulation were rescued separately through interfering miR-133b and HOXB8, suggesting AR/ZEB1-AS1/miR-133b/HOXB8 exerted crucial functions in tumorigenesis and progression of CCA.

## INTRODUCTION

Cholangiocarcinoma (CCA) is a highly aggressive malignant cancer deriving from bile duct epithelial cells. The incidence of CCA is gradually rising, and prognosis of patients remains quite poor [1]. CCA patients in early stage are usually asymptomatic and also lack sensitive early diagnostic indicators. These reasons lead to most advanced patients. Radical resection is the only effective way to thoroughly cure CCA, while this is only for early patients without metastasis. Besides, current chemotherapy and radiotherapy can not achieve satisfactory therapeutic effect [2]. Finding efficient early diagnostic biomarkers and therapeutic targets is urgently needed to improve the long-term survival of patients with CCA. Hence, exploring pivotal molecular abnormality involved in malignant progression of CCA should be prioritized.

Thanks to recent advances in genome sequencing technique, noncoding RNAs have drawn widespread attention [3]. Long noncoding RNAs (lncRNAs) represent a type of noncoding RNAs with longer than 200 nucleotides. They are characterized by no protein-coding ability due to lack an obvious open reading frame (ORF) [4]. Accumulating evidence manifests that lncRNAs are involved in regulation of gene expression at epigenetic, transcriptional and post-transcriptional levels. They participate in pathological processes of multifarious diseases through the mechanisms of competitive endogenous RNA (ceRNA), regulatory signal, protein scaffold, transcript decoy, and transcript guide [5–7]. Many lncRNAs are also corroborated to act as oncogenes or tumor suppressors in CCA progression. Aberrantly expressed lncRNAs can predict a poor prognosis and promote malignant phenotypes of CCA cells, suggesting the potential clinical value of these RNAs [8].

Among all the cancer-related lncRNAs, zinc finger E-box binding homeobox 1 antisense 1 (ZEB1-AS1) is a well-characterized oncogenic lncRNA. ZEB1-AS1 is located at chromosome 10p11.22 region in physical contiguity with ZEB1 [9]. ZEB1-AS1 is an antisense transcript originating from promoter of ZEB1, which is a prominent transcription factor in relation to tumor metastasis [10]. ZEB1-AS1 was originally discovered to be overexpressed in esophageal squamous cell carcinoma [11]. Subsequently, ZEB1-AS1 dysregulation was confirmed in varieties of digestive system cancers, including hepatocellular carcinoma, gastric cancer, and colorectal cancer [9, 13, 14]. For instance, ZEB1-AS1 overexpression was remarkably related to worse survival time, and it represented an unfavorable independent prognostic factor in gastric cancer patients [12]. In colorectal cancer, ZEB1-AS1 facilitated cell proliferation and migration through regulating miR-101/ZEB1 [13]. However, the biological function of ZEB1-AS1 in occurrence and development of cholangiocarcinoma remains unclear.

In the present study, we first confirmed that ZEB1-AS1 expression was markedly upregulated in CCA and related to lymph node invasion, advanced TNM stage and poor survival. Upregulated ZEB1-AS1 was an independent risk factor for prognosis of CCA patients. In addition, ZEB1-AS1 was induced by transcription factor androgen receptor (AR). ZEB1-AS1 contributed to cellular malignant phenotypes by the mechanism of directly modulating miR-133b/homeobox B8 (HOXB8). Taken together, ZEB1-AS1 functions as a tumor-promoting lncRNA in CCA progression, and ZEB1-AS1 is expected to be a valuable tumor biomarker or intervention target.

## RESULTS

### Expression level and clinical significance of ZEB1-AS1 in CCA

**Figure 1:**
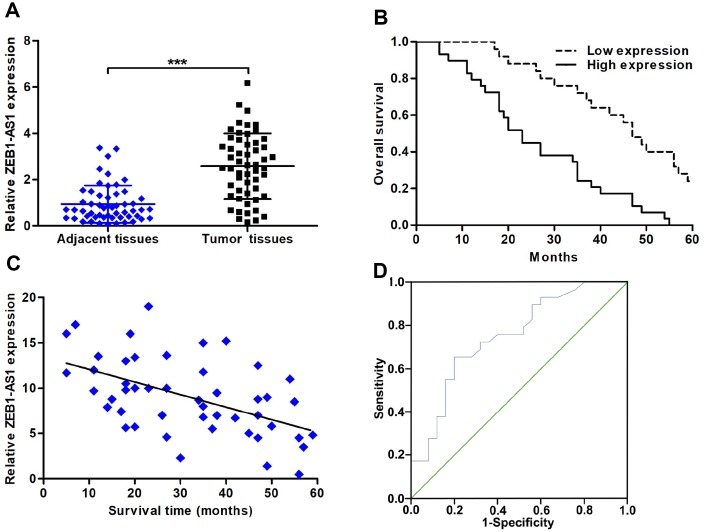
**The expression of ZEB1-AS1 and its correlation with clinicopathological characteristics and prognosis**. (**A**) ZEB1-AS1 expression in CCA tissues and paired adjacent nontumor bile duct tissues was detected by qRT-PCR. (**B**) CCA patients were divided into two groups according to average value of ZEB1-AS1 expression. Overall survival was evaluated between high and low ZEB1-AS1 expression groups by using Kaplan-Meier method and log-rank test. (**C**) The correlation between relative ZEB1-AS1 expression and survival time of CCA patients was assessed by Pearson correlation analysis. (**D**) The sensitivity and specificity of ZEB1-AS1 as a prognostic marker were analyzed by ROC curve. ^***^P < 0.001.

The expression of ZEB1-AS1 was detected in CCA tissues by using quantitative real-time polymerase chain reaction (qRT-PCR). Results showed that ZEB1-AS1 was significantly overexpressed in CCA tissues compared with that in paired adjacent nontumor bile duct tissues (Figure 1A). Based on this results, ZEB1-AS1 was likely involved in pathological process of CCA. Accordingly, we analysed the correlation between ZEB1-AS1 expression and clinicopathological characteristics of CCA patients through dividing all cases into high ZEB1-AS1 expression group and low ZEB1-AS1 expression group. Results displayed that increased ZEB1-AS1 was significantly associated with lymph node invasion and advanced TNM stage. Nevertheless, ZEB1-AS1 expression was not related to other clinicopathological parameters in this study (Table 1). Survival correlation was also analysed by means of Kaplan-Meier, and the findings revealed that patients with high ZEB1-AS1 expression had worse overall survival (OS) than those with low ZEB1-AS1 expression (log rank *P* < 0.001; Figure 1B). Furthermore, Pearson correlation analysis demonstrated that ZEB1-AS1 expression was negatively related to survival time of CCA patients (*r* = -0.5202, *P* < 0.001; Figure 1C). Above results illustrated that ZEB1-AS1 might work as a biomarker in CCA. Therefore, we further evaluated prognostic value of ZEB1-AS1 in CCA patients via univariate and multivariate analyses. As shown in Table 2, upregulated ZEB1-AS1 expression, advanced TNM stage, and lymph node invasion were closely correlated with survival of CCA patients. Among these parameters, upregulated ZEB1-AS1 expression and advanced TNM stage were confirmed to be independent unfavorable prognostic factors of CCA patients. For prognostic efficiency of ZEB1-AS1, receiver operating characteristic curve (ROC) analysis showed that value of area under curve (AUC) was 0.749 (95% CI: 0.618-0.880) with 65.5% sensitivity and 80.0% specificity (*P* < 0.001; Figure 1D).

**Table 1:** Correlation between ZEB1-AS1 expression and clinicopathological characteristics of CCA patients.

**Clinicopathological parameters**	**Total cases (*n* = 54)**	**ZEB1-AS1 expression**		***P*-value**
		**Low (*n* = 25)**	**High (*n* = 29)**	
Age (years)				
< 60	17	9	8	0.507
≥ 60	37	16	21	
Gender				
Male	23	10	13	0.721
Female	31	15	16	
Tumor location				
Intrahepatic	16	9	7	0.341
Extrahepatic	38	16	22	
Histological type				
Adenocarcinoma	49	24	25	0.216
Non-adenocarcinoma	5	1	4	
Differentiation grade				
Well/moderate	22	13	9	0.118
Poor/undifferentiated	32	12	20	
TNM stage				
I-II	24	16	8	0.007^**^
III-IV	30	9	21	
Lymph node invasion				
Yes	35	12	23	0.016^*^
No	19	13	6	
Serum CA19-9 level				
> 37 U/ml	34	13	21	0.121
≤ 37 U/ml	20	12	8	

Note. ^*^P < 0.05, ^**^P < 0.01. ZEB1-AS1, Zinc finger E-box binding homeobox 1 antisense 1; CCA, cholangiocarcinoma.

**Table 2:** Univariate and multivariate analyses for overall survival of CCA patients.

**Variables**	**Univariate analysis**			**Multivariate analysis**		
	**HR**	**95% CI**	***P*-value**	**HR**	**95% CI**	***P*-value**
Age (years)						
≥ 60 *vs*. < 60	1.365	0.741-2.517	0.318			
Gender						
Male *vs*. Female	1.248	0.695-2.239	0.458			
Tumor location						
Extrahepatic *vs*. Intrahepatic	1.313	0.694-2.486	0.402			
Histological type						
Adenocarcinoma vs. Non-adenocarcinoma	1.366	0.537-3.473	0.513			
Differentiation grade						
Poor/undifferentiated *vs*. Well/moderate	1.646	0.910-2.978	0.099			
Serum CA19-9 level						
> 37 U/ml *vs.* ≤ 37 U/ml	1.486	0.819-2.697	0.193			
TNM stage						
III-IV *vs*. I-II	2.024	1.132-3.620	0.017^*^	2.193	1.223-3.932	0.008^**^
Lymph node invasion						
Yes *vs*. No	1.884	1.025-3.463	0.041^*^	1.784	0.959-3.318	0.067
ZEB1-AS1 expression						
Low *vs*. High	2.220	1.202-4.101	0.011^*^	2.569	1.355-4.870	0.004^**^

Note. ^*^P < 0.05, ^**^P < 0.01. CCA, cholangiocarcinoma; HR, hazard ratio; CI, confidence interval; ZEB1-AS1, Zinc finger E-box binding homeobox 1 antisense 1.

### Upregulated ZEB1-AS1 promoted cellular viability and stemness in CCA

**Figure 2:**
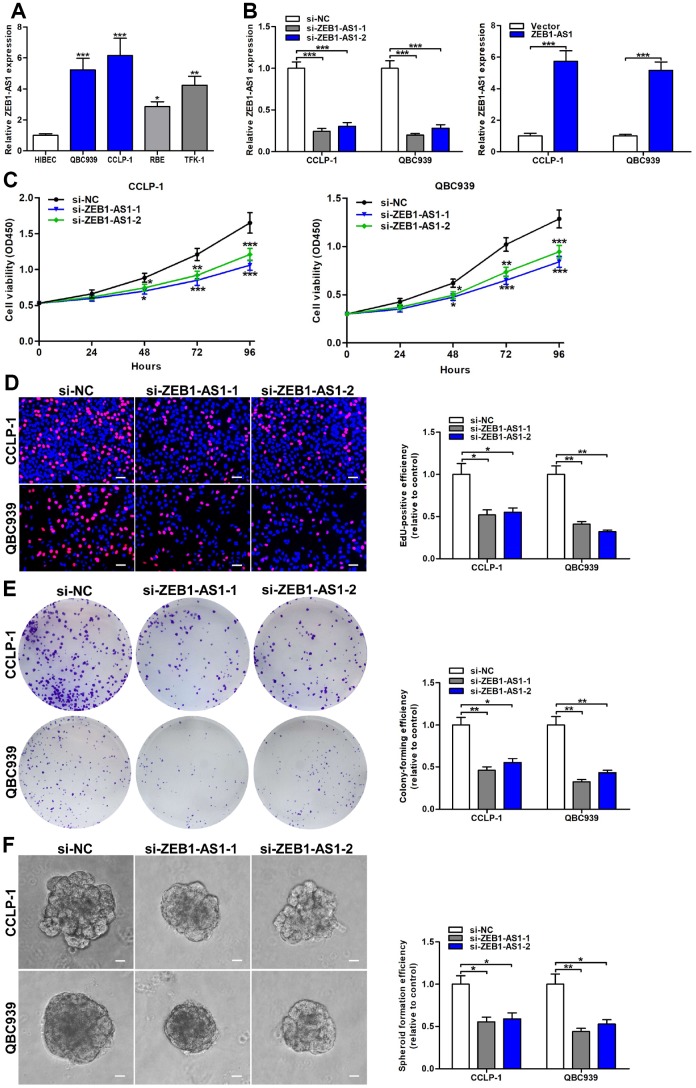
**Increased ZEB1-AS1 facilitated the viability and stemness of CCA cells.** (**A**) The expression of ZEB1-AS1 in CCA QBC939, CCLP-1, RBE, TFK-1 cells and normal HIBEC. (**B**) The knockdown efficiencies of si-ZEB1-AS1-1 and si-ZEB1-AS1-2 as well as amplification efficiency of pcDNA3.1-ZEB1-AS1 were monitored through qRT-PCR. (**C**) CCK-8 proliferation curves were drawn to show the effect of ZEB1-AS1 on cellular proliferation. (**D**) The red stains representing proliferative activity were reduced in QBC939 and CCLP-1 cells transfected with si-ZEB1-AS1-1 and si-ZEB1-AS1-2. (**E**) The cell colonies were decreased in si-ZEB1-AS1 cells revealed by colony formation assays. (**F**) Spheroid forming abilities of QBC939 and CCLP-1 cells transfected with si-ZEB1-AS1-1 and si-ZEB1-AS1-2 were restrained. ^*^P < 0.05, ^**^P < 0.01, ^***^P < 0.001.

Based on ZEB1-AS1 expression in CCA tissues, we further detected ZEB1-AS1 in CCA cells via qRT-PCR. Results confirmed that ZEB1-AS1 was also overexpressed in QBC939, CCLP-1, RBE and TFK-1 compared with that in HIBEC (Figure 2A). According to qRT-PCR results, QBC939 and CCLP-1 were selected for further study. After transfection, ZEB1-AS1 expression was markedly amplified or knocked down in QBC939 and CCLP-1 cells (Figure 2B). For assessing proliferative ability, we performed cell counting kit-8 (CCK-8) assay. The proliferation curves displayed that knocking down ZEB1-AS1 inhibited proliferation of CCA cells in comparison with controls (Figure 2C). 5-ethynyl-2’-deoxyuridine (EdU) incorporation assay was also carried out to measure cellular viability, and proliferation activity was suppressed in si-ZEB1-AS1 cells (Figure 2D). Afterwards, we detected clonogenic capacity of CCA cells through performing colony formation assay. As displayed in Figure 2E, silencing ZEB1-AS1 restrained colony-forming ability of QBC939 and CCLP-1 cells. More importantly, spheroid formation assay verified that ZEB1-AS1 knockdown attenuated tumor stemness in CCA cells (Figure 2F). To sum up, these findings illustrated that ZEB1-AS1 promoted malignant proliferation and tumor stemness in CCA.

**Increased ZEB1-AS1 facilitated cellular migration and invasion by promoting epithelial-mesenchymal transition (EMT)**

**Figure 3:**
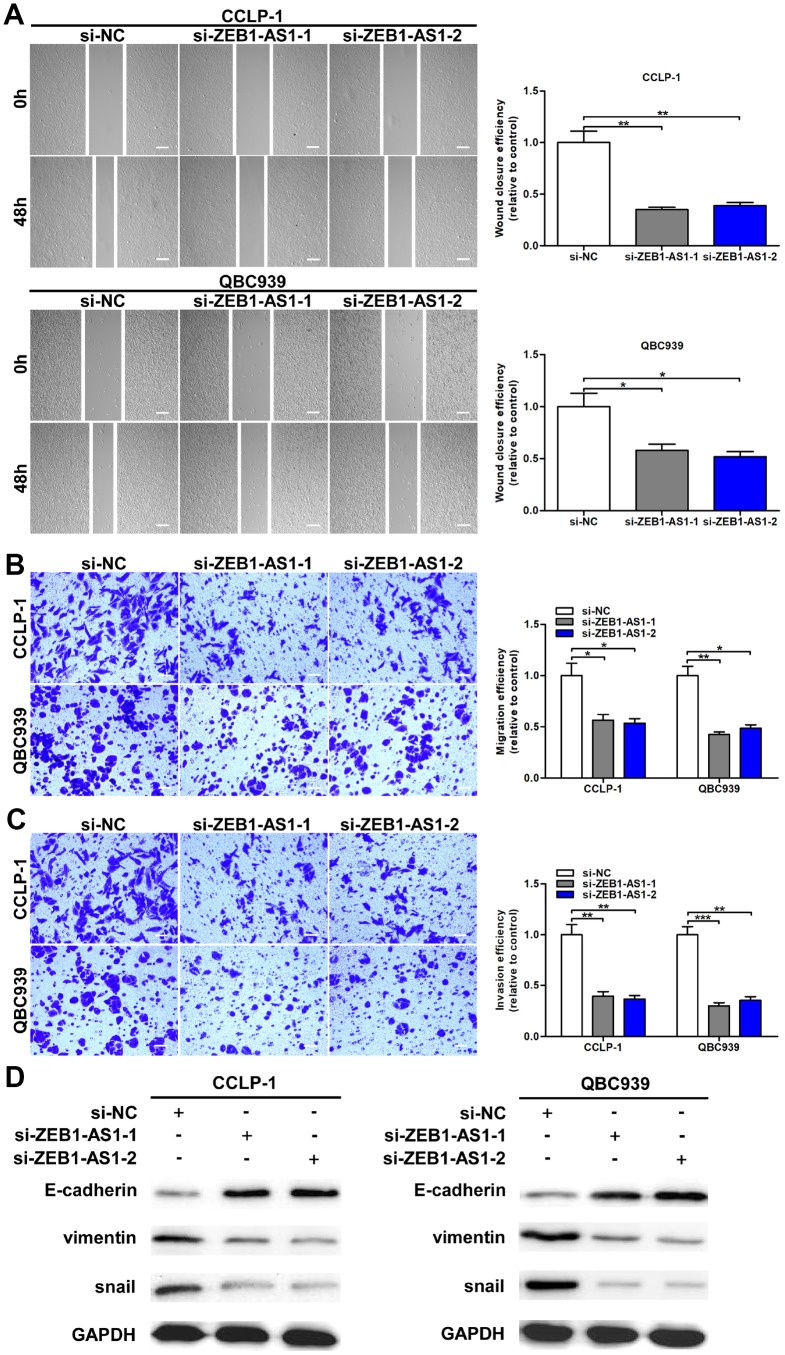
**Upregulated ZEB1-AS1 promoted cellular migration and invasion through promoted EMT process.** (**A**) The wound closure of QBC939 and CCLP-1 cells transfected with si-ZEB1-AS1-1 and si-ZEB1-AS1-2 was delayed verified by wound healing assays. (**B**) The numbers of migrating cells were decreased in si-ZEB1-AS1 cells. (**C**) Transwell assays displayed that knocking down ZEB1-AS1 inhibited invasion of QBC939 and CCLP-1 cells compared with controls. (**D**) EMT-related proteins including epithelial marker (E-cadherin) and mesenchymal markers (snail and vimentin) were measured via western blot. ^*^P < 0.05, ^**^P < 0.01, ^***^P < 0.001.

As confirmed by wound healing assay, cellular motility was repressed by knocking down ZEB1-AS1 in CCA cells (Figure 3A). Moreover, transwell assay was conducted to investigate metastatic ability of CCA cells in this study. As shown in Figure 3B, silencing ZEB1-AS1 reduced migratory numbers of CCA cells demonstrated through transwell assay without Matrigel. Subsequently, we confirmed that ZEB1-AS1 knockdown caused a decrease of invasive cells in transwell assay with Matrigel (Figure 3C). Studies confirmed that tumor metastasis mainly depended on EMT process [14]. Accordingly, we measured the epithelial and mesenchymal markers of EMT in QBC939 and CCLP-1 cells via western blot. Results confirmed that knocking down ZEB1-AS1 decreased snail and vimentin expression, whereas E-cadherin expression was increased in CCA cells (Figure 3D). In summary, ZEB1-AS1 contributed to migration and invasion of CCA cells in part by promoting EMT.

### AR directly promoted ZEB1-AS1 expression through acting as a transcription factor

**Figure 4:**
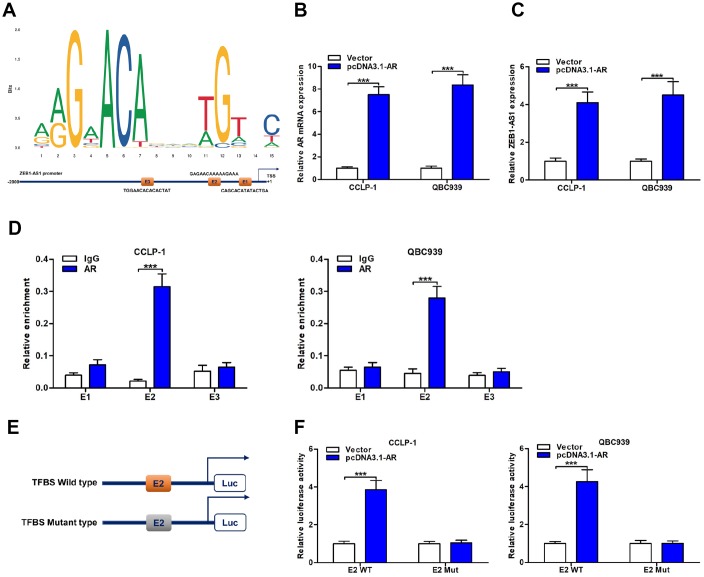
**ZEB1-AS1 was induced by transcription factor AR.** (**A**) AR sequence and binding sites (E1, E2 and E3) to ZEB1-AS1 promoter region were predicted by using JASPAR database (http://jaspar.genereg.net/). (**B**) The pcDNA3.1-AR amplified AR mRNA expression in QBC939 and CCLP-1 cells compared with empty vector. (**C**) Upregulated AR facilitated ZEB1-AS1 expression in QBC939 and CCLP-1 cells corroborated by qRT-PCR. (**D**) ChIP assays were performed to confirm the direct binding of AR to ZEB1-AS1 promoter in QBC939 and CCLP-1 cells. (**E**) Luciferase reporter plasmids were constructed with TFBS E2, including wild type and mutant type. (**F**) The luciferase activity of TFBS E2 WT was markedly promoted by pcDNA3.1-AR cotransfection compared with controls in QBC939 and CCLP-1 cells. ^***^P < 0.001.

To explore upstream regulatory mechanism of ZEB1-AS1, we screened possible transcriptional regulators of ZEB1-AS1 promoter region by using JASPAR database (http://jaspar.genereg.net/). As displayed in Figure 4A, AR as a transcription factor was identified to directly bind to ZEB1-AS1 promoter including three transcription factor binding site (TFBS; E1, E2 and E3). Accordingly, we decided to explore function of AR on ZEB1-AS1 via basic experiments. First of all, CCA cells were separately transfected with pcDNA3.1-AR and empty vector. Results displayed that pcDNA3.1-AR not only amplified AR but also increased ZEB1-AS1 expression (Figure 4B, 4C). Subsequently, chromatin immunoprecipitation (ChIP) assay uncovered that TFBS E2 fragments were significantly recruited by AR antibody (Figure 4D), suggesting that this region was a direct target of AR. Furthermore, E2 with wild type or mutant type was separately cloned into luciferase reporter plasmids (Figure 4E), and this assay further confirmed the direct binding of AR to TFBS E2 (Figure 4F). Taken together, AR was a direct upstream inducer of ZEB1-AS1 in CCA.

### Prediction of a ceRNA pathway regarding ZEB1-AS1/miR-133b/HOXB8

**Figure 5:**
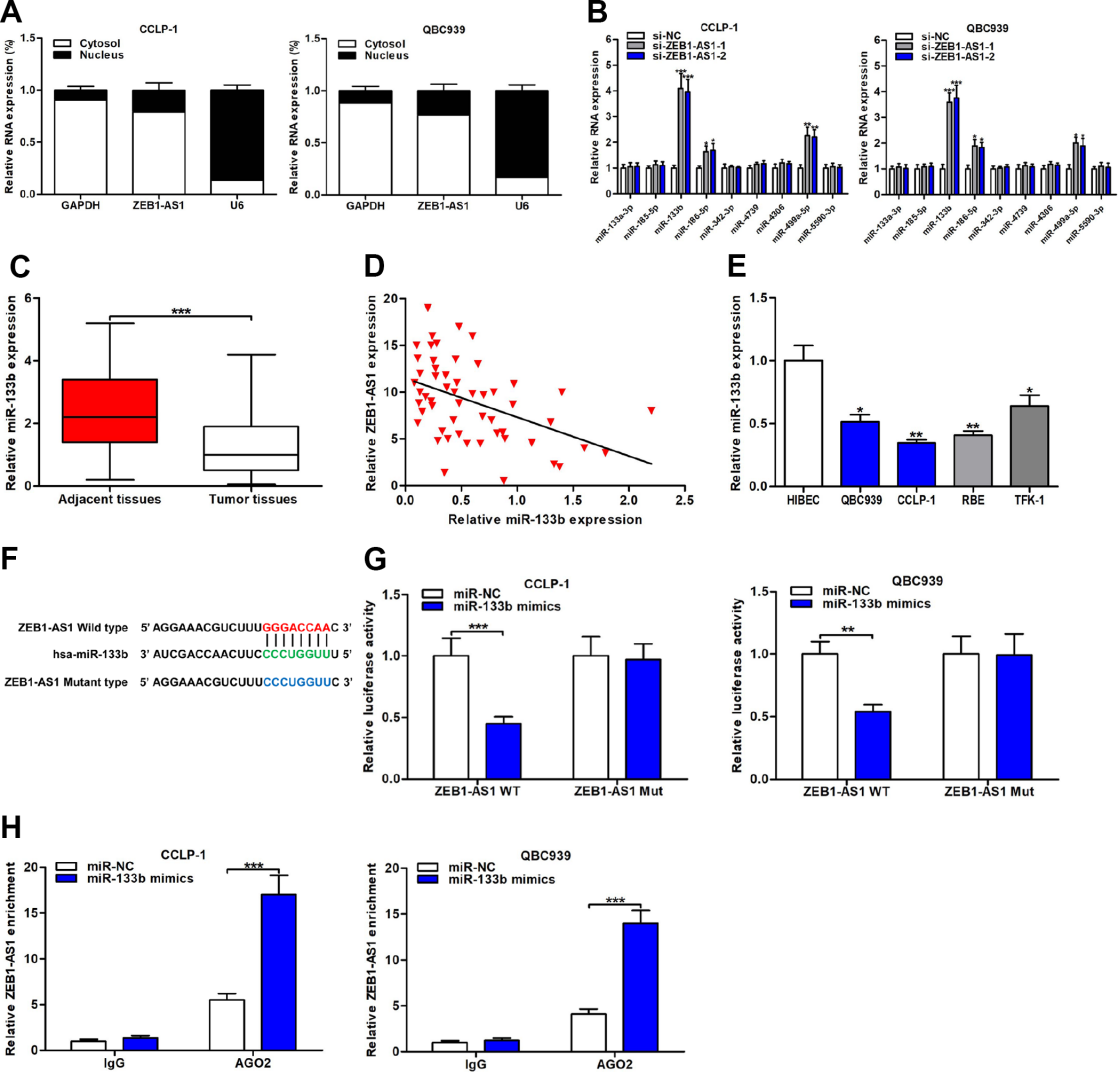
**ZEB1-AS1 functioned as a sponge for miR-133b in CCA cells.** (**A**) Subcellular localization of ZEB1-AS1 was tested by subcellular fractionation assays. GAPDH and U6 were used as endogenous controls for cytoplasm and nucleus, respectively. (**B**) The expression levels of predicted miRNAs were detected after knocking down ZEB1-AS1 in QBC939 and CCLP-1 cells. (**C**) The expression of miR-133b in CCA tissues and paired adjacent nontumor bile duct tissues. (**D**) The correlation between relative ZEB1-AS1 expression and relative miR-133b expression in CCA tissues. (**E**) The miR-133b expression in CCA cells (QBC939, CCLP-1, RBE, TFK-1) and normal HIBEC. (**F**) Luciferase reporter plasmids were constructed with miR-133b-binding site region of ZEB1-AS1 sequence, including wild type and mutant type. (**G**) Luciferase reporter assays showed that cotransfected miR-133b mimics significantly inhibited luciferase activity of ZEB1-AS1 wild type. (**H**) AGO2 RIP assays further suggested the binding of miR-133b to ZEB1-AS1. ^*^P < 0.05, ^**^P < 0.01, ^***^P < 0.001.

The localization of molecules in cells generally determined the pathway by which they functioned. Thus, we measured ZEB1-AS1 location in CCA cells through subcellular fractionation assay. Results showed that ZEB1-AS1 was mainly expressed in cytoplasm compared to nucleus (Figure 5A), implying ZEB1-AS1-induced mechanisms predominantly at post-transcriptional level. Besides, mounting studies have indicated that lncRNAs regulated downstream targets relying on ceRNA mode. Hence, we evaluated ZEB1-AS1-induced mechanisms according to ceRNA mode in the online database StarBase v3.0 (https://bio.tools/starbase). Predicted results showed that ZEB1-AS1 increased oncogene HOXB8 by competitively binding to many miRNAs, and miR-133b was chosen for further study according to the qRT-PCR analysis (Figure 5B).

### ZEB1-AS1 inhibited miR-133b by functioning as a sponge

Expression of miR-133b was monitored in CCA tissues and result displayed that miR-133b was downexpressed in CCA tissues compared with the controls (Figure 5C). Correlation analysis corroborated that miR-133b expression was negatively related to ZEB1-AS1 expression in CCA tissues (*r* = -0.4622, *P* < 0.001; Figure 5D). In addition, miR-133b expression was also assessed in CCA cells, and the result was consistent with detection in tumor tissues (Figure 5E). Subsequently, dual luciferase reporter gene assays were conducted to demonstrate the binding of ZEB1-AS1 to miR-133b through cloning miR-133b-binding site region of ZEB1-AS1 sequence into luciferase reporter plasmids, including ZEB1-AS1 wild type and mutant type (Figure 5F). As expected, upregulated miR-133b caused by cotransfected with miR-133b mimics suppressed luciferase activity of ZEB1-AS1 wild type rather than mutant type (Figure 5G), suggesting that ZEB1-AS1 bound to miR-133b through the binding site. RNA immunoprecipitation (RIP) assays were performed for further verifying their target binding. Results displayed that ZEB1-AS1 enrichment was higher in Argonaute 2 (AGO2) groups with miR-133b mimics than controls (Figure 5H). Above findings elucidated that ZEB1-AS1 inhibited miR-133b by serving as a sponge in CCA cells.

### HOXB8 was a direct target of miR-133b in CCA cells

**Figure 6:**
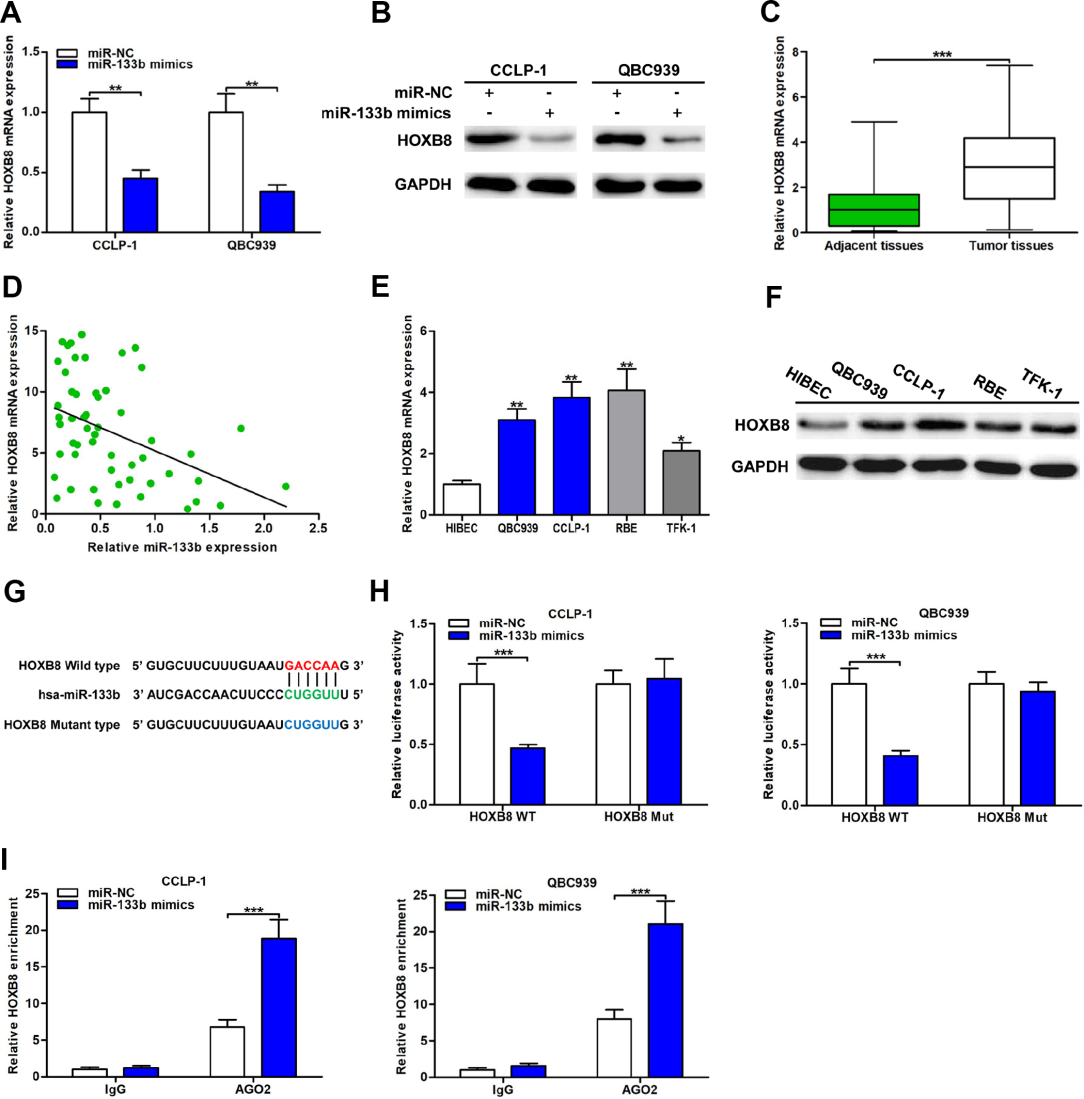
**MiR-133b was a direct regulator of HOXB8 in CCA cells.** (**A**) MiR-133b restrained HOXB8 mRNA expression confirmed by qRT-PCR. (**B**) MiR-133b refrained HOXB8 protein expression testified via western blot in QBC939 and CCLP-1 cells. (**C**) The expression of HOXB8 mRNA in CCA tissues and paired adjacent nontumor bile duct tissues. (**D**) The correlation between relative HOXB8 mRNA expression and relative miR-133b expression in CCA tissues. (**E**) The HOXB8 mRNA expression in QBC939, CCLP-1, RBE, TFK-1 and normal HIBEC. (**F**) The HOXB8 protein expression in CCA cells (QBC939, CCLP-1, RBE, TFK-1) and normal HIBEC. (**G**) Luciferase reporter plasmids were constructed with miR-133b-binding site region of HOXB8 sequence, including wild type and mutant type. (**H**) The luciferase activity of HOXB8 wild type was inhibited by miR-133b mimics cotransfection. (**I**) AGO2 RIP assays were conducted to further demonstrate the binding of miR-133b to 3’UTR of HOXB8. ^*^P < 0.05, ^**^P < 0.01, ^***^P < 0.001.

The qRT-PCR analysis revealed that upregulated miR-133b restrained HOXB8 expression both at mRNA and protein levels (Figure 6A, 6B). HOXB8 mRNA expression was markedly increased in CCA tissues and inversely related to miR-133b expression (*r* = -0.4227, *P* < 0.01; Figure 6C, 6D). Furthermore, both mRNA and protein of HOXB8 were overexpressed in CCA cells (Figure 6E, 6F). Luciferase reporter assays confirmed that upregulated miR-133b repressed luciferase activity of HOXB8 wild type, while luciferase activity of HOXB8 mutant type was not significantly affected (Figure 6G, 6H). In addition, AGO2 RIP assays further confirmed that miR-133b enriched HOXB8 mRNA in CCA cells (Figure 6I). Overall, miR-133b repressed HOXB8 in CCA cells by interacting with the binding site of HOXB8 3’UTR.

### AR-induced ZEB1-AS1 facilitated CCA progression by regulating miR-133b/HOXB8

**Figure 7:**
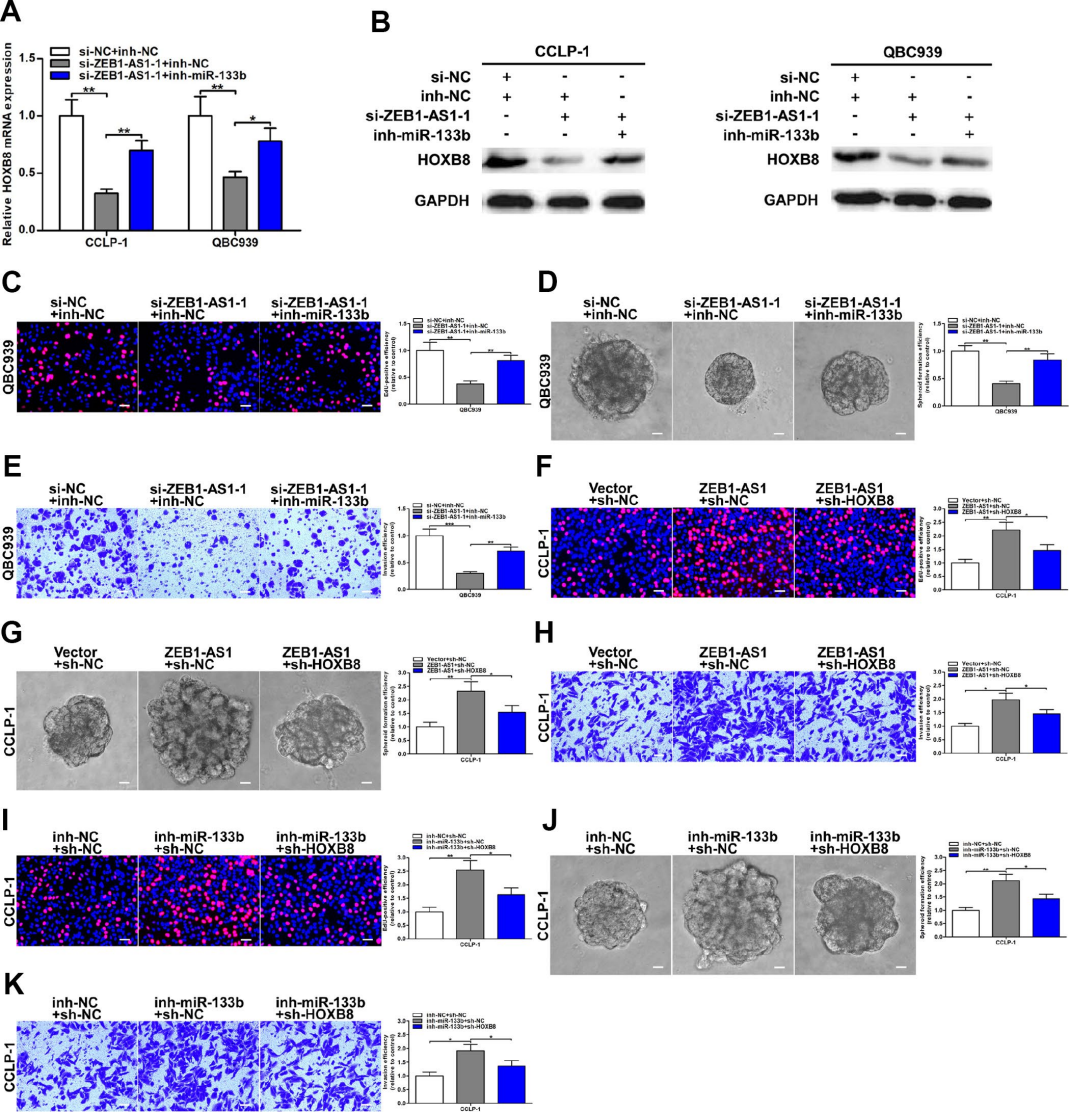
**ZEB1-AS1 promoted malignant progression of CCA through mediating miR-133b/HOXB8.** (**A**, **B**) HOXB8 downexpression caused by ZEB1-AS1 knockdown was saved by silencing miR-133b. (**C–E**) Rescue assays of EdU, spheroid formation and transwell confirmed that inhibition of proliferation, stemness and invasion induced by knocking down ZEB1-AS1 was saved through silencing miR-133b, respectively. (**F–H**) CCLP-1 cells cotransfected with pcDNA3.1-ZEB1-AS1 and sh-HOXB8 were used to carry out EdU, spheroid formation and transwell assays, respectively. (**I–K**) Restoration of HOXB8 expression rescued the promotion of proliferation, stemness and invasion generated through miR-133b knockdown in EdU, spheroid formation and transwell assays, respectively. ^*^P < 0.05, ^**^P < 0.01, ^***^P < 0.001.

To further determine the regulatory relationship between ZEB1-AS1 and HOXB8, rescue experiments were carried out. Results uncovered that knocking down ZEB1-AS1 depressed HOXB8 expression both at mRNA and protein levels, and then inhibition of HOXB8 was partially saved through silencing miR-133b (Figure 7A, 7B). These data illuminated that AR-induced ZEB1-AS1 as a ceRNA competitively bound miR-133b to increase HOXB8 in CCA cells. Next, rescue experiments were also performed to demonstrate whether ZEB1-AS1 promoted malignant biological behavior of CCA cells by regulating miR-133b and HOXB8. As displayed in Figure 7C, EdU rescue assay verified that reduction of proliferation caused by ZEB1-AS1 knockdown was saved by silencing miR-133b. Spheroid formation assay also suggested that silencing miR-133b partly rescued suppression of tumor stemness caused through knocking down ZEB1-AS1 (Figure 7D). In addition, recovery of miR-133b saved invasive inhibition generated via silencing ZEB1-AS1 (Figure 7E). These results indicated that ZEB1-AS1 contributed to CCA development in part by inhibiting miR-133b. Subsequently, the rescue assays of proliferation, tumor stemness, and invasion also testified that cancer-promoting effect caused by ZEB1-AS1 overexpression was saved by knocking down HOXB8 (Figure 7F–7H), indicating that ZEB1-AS1 promoted malignant progression of CCA partly by promoting HOXB8. Furthermore, tumor-promoting function generated through silencing miR-133b was saved by knocking down HOXB8 confirmed by the rescue assays of EdU, spheroid formation and transwell (Figure 7I–7K). Taken together, AR-induced ZEB1-AS1 promoted CCA development partly by regulating miR-133b/HOXB8.

### ZEB1-AS1/miR-133b/HOXB8 promoted CCA tumorigenesis *in vivo*

**Figure 8:**
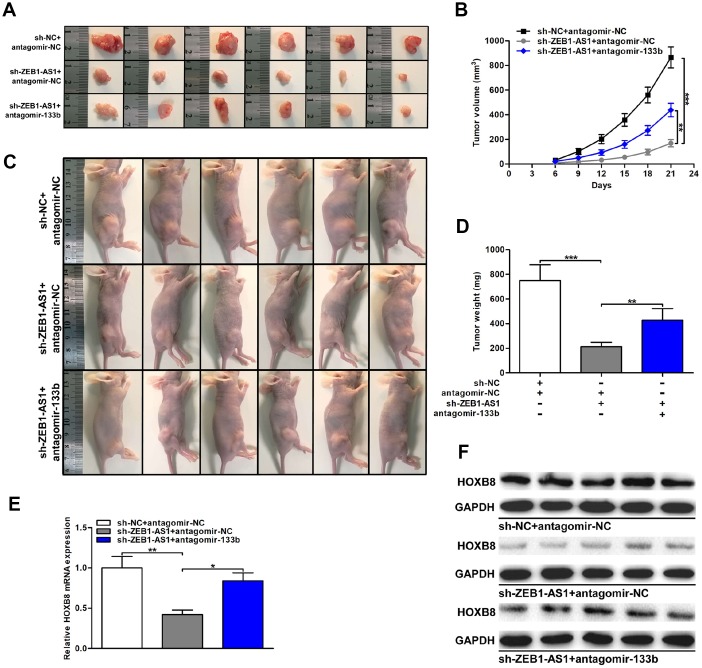
**ZEB1-AS1/miR-133b/HOXB8 contributed to CCA tumorigenesis *in vivo*.** (**A**) Xenograft tumors were resected on the 21^st^ day after injection. (**B**) Tumor volumes were calculated every 3 days throughout the course of tumor growth. (**C**) CCLP-1 cells cotransfected with sh-ZEB1-AS1 and antagomir-133b were subcutaneously injected into the posterior flanks of mice. (**D**) Tumor weights were measured after excision. (**E**, **F**) HOXB8 expression in xenograft tumors of the three groups (sh-NC/antagomir-NC, sh-ZEB1-AS1/antagomir-NC, sh-ZEB1-AS1/antagomir-133b). ^*^P < 0.05, ^**^P < 0.01, ^***^P < 0.001.

Finally, the effect of ZEB1-AS1/miR-133b/HOXB8 in nude mice was investigated via subcutaneous tumor formation experiments. As shown in Figure 8A–8D, knocking down ZEB1-AS1 not only depressed tumor volumes throughout the course of cancer growth, but also remarkably inhibited tumor weights. Notably, the inhibitory effect caused by silencing ZEB1-AS1 was partly rescued through miR-133b knockdown. HOXB8 expression in xenograft tumors was detected via qRT-PCR and western blot. Results displayed that HOXB8 expression was restrained both at mRNA and protein levels by silencing ZEB1-AS1. More importantly, restoration of miR-133b expression saved the downexpression of HOXB8 generated through ZEB1-AS1 knockdown (Figure 8E, 8F). These findings indicated that ZEB1-AS1 promoted tumor growth of CCA by regulating miR-133b/HOXB8 not only *in vitro* but also *in vivo*.

## DISCUSSION

The discovery of lncRNAs enriches our understanding of the complexity and diversity of genetic information at the transcriptional level and provides a new research direction for improving the diagnosis and treatment of malignancies [15]. Several lncRNAs have been found to exhibit pivotal regulatory functions in occurrence and development of cholangiocarcinoma. For example, Sox2 overlapping transcript (Sox2ot) was overexpressed in CCA and related to prognosis of patients [16]; small nucleolar RNA host gene 1 (SNHG1) facilitated proliferation and migration of CCA cells through epigenetically inhibiting cyclin dependent kinase inhibitor 1A (CDKN1A) [17]. ZEB1-AS1 is a promising lncRNA that has been discovered to function in multiple tumors, such as hepatocellular carcinoma, gastric cancer, colorectal cancer, and esophageal cancer [9, 11–13]. Nevertheless, functions of ZEB1-AS1 in cholangiocarcinoma are not explored before. High-efficiency prognostic markers are extremely crucial for monitoring long-term survival of cancer patients. Studies confirmed that patients with increased ZEB1-AS1 possessed worse overall survival and disease-free survival in esophageal cancer [11]; and upregulated ZEB1-AS1 was an independent poor prognostic factor of patients with hepatocellular carcinoma [9]. The present study confirmed upregulated ZEB1-AS1 expression in CCA tissues and cells compared with the controls, respectively. Moreover, upregulated ZEB1-AS1 was significantly associated with advanced TNM stage, lymph node invasion and poor survival. More importantly, ZEB1-AS1 was demonstrated to be a sensitive and specific marker for prognosis of CCA patients. These results illustrate that ZEB1-AS1 plays an oncogenic role in CCA and exhibits excellent features to be a tumor biomarker for CCA patients.

The influence of ZEB1-AS1 on malignant biological behavior of CCA was testified in this study. CCK-8 and EdU assays verified that knocking down ZEB1-AS1 restrained tumor cell proliferation. Clonogenic ability was also suppressed in si-ZEB1-AS1 cells indicated by colony formation assay. Besides, spheroid formation assay verified that silencing ZEB1-AS1 repressed tumor stemness in CAA cells. For *in vivo* experiment, we verified that ZEB1-AS1 knockdown repressed both volume and weight of tumor xenograft. These data illustrate that ZEB1-AS1 contributes to CCA cell viability both *in vitro* and *in vivo*. Pertinent to tumor metastasis, wound healing and transwell assays documented that silencing ZEB1-AS1 inhibited migration and invasion of CCLP-1 and QBC939 cells. In addition, silencing ZEB1-AS1 reversed EMT, which represented an indispensable process in tumor metastasis [18], through increasing epithelial markers and decreasing mesenchymal markers. These findings elucidate that ZEB1-AS1 promotes migration and invasion of CCA cells partly by promoting EMT process. Overall, ZEB1-AS1 is involved in CCA progression and facilitates cellular proliferation, stemness and metastasis.

To investigate upstream and downstream regulatory mechanisms of ZEB1-AS1 in CCA development, bioinformatics databases were used to predict potential targets. AR is a ligand-sensitized transcription factor that binds to promoter region of target gene by its DNA-binding domain [19]. Huang et al. revealed that AR regulated TMPO antisense RNA 1 (TMPO-AS1) transcription through working as a transcription factor [20]. In this study, AR was uncovered to activate ZEB1-AS1 by binding to the promoter region around E2, suggesting that AR is an upstream modulator of ZEB1-AS1. CeRNA pathway is a main regulatory method of noncoding RNAs, by which lncRNAs competitively bind miRNAs to restrain or degrade them, thereby releasing downstream targets of miRNAs [21]. Qian et al. uncovered that ZEB1-AS1 promoted pulmonary fibrosis through competitively binding miR-141-3p to increase ZEB1 [22]. MiR-133b was confirmed to be a tumor suppressor in various cancers, such as hepatocellular carcinoma, gastric cancer, and colorectal cancer [23–25]. On the contrary, HOXB8 has been found to promote tumor progression in many malignancies, including gastric cancer, colorectal cancer, ovarian serous carcinoma, and osteosarcoma [26–29]. In the present study, we demonstrated that ZEB1-AS1 increased HOXB8 by sponging miR-133b, thereby facilitating CCA development. These results indicate that AR/ZEB1-AS1/miR-133b/HOXB8 pathway performs pivotal functions in occurrence and development of CCA.

In conclusion, our study demonstrated that ZEB1-AS1 was overexpressed in CCA and significantly related to clinicopathological characteristics and poor survival. Furthermore, upregulated ZEB1-AS1 was an independent poor prognostic marker with high sensitivity and specificity. In addition, AR-induced ZEB1-AS1 promoted proliferation, stemness, metastasis and EMT process by functioning as a ceRNA to regulate miR-133b/HOXB8. Accordingly, ZEB1-AS1 is expected to reach the clinic to be a valuable tumor biomarker or therapeutic target.

## MATERIALS AND METHODS

### Tissue specimens and clinical data

This study was approved by the Ethics Committee of Second Affiliated Hospital of Harbin Medical University (KY2018-354). All the patients in this study signed written informed consent. CCA tissues and paired adjacent nontumor bile duct tissues were collected from 54 patients undergoing surgical operation from 2010 to 2012 at Second Affiliated Hospital of Harbin Medical University. The tissue samples were immediately frozen and stored in liquid nitrogen after excision. Two professional pathologists were required to authenticate all specimens. Patients who used radiotherapy and chemotherapy before surgery had been excluded in this study.

### Cell lines and transfection

One normal human biliary epithelial cell HIBEC and four human CCA cell lines (QBC939, CCLP-1, RBE, TFK-1) were used in this study. RBE was purchased from the Cell Bank of Chinese Academy of Sciences (Shanghai, China), and other cell lines were preserved in our laboratory. Dulbecco’s modified Eagle’s medium (DMEM) and Roswell Park Memorial Institute-1640 (RPMI-1640) supplemented with 10% fetal bovine saline (FBS; Invitrogen, Carlsbad, CA) were utilized to culture the cells in a humidified condition with 5% CO_2_ and 37^°^C. Besides, all cultured cells were passed for less than six months. For knockdown and overexpression of genes, small interfering RNA (siRNA)/si-negative control (NC), short hairpin RNA (shRNA)/sh-NC, antagomir/antagomir-NC, inhibitor/inh-NC, mimics/miR-NC and pcDNA3.1/pcDNA3.1-NC (GenePharma, Shanghai, China) were acquired for transfection by applying Lipofectamine 3000 (Invitrogen) according to the manufacturer’s directions. After interaction of 48 h, the transfected cells were harvested. The qRT-PCR and western blot were utilized to assess the knockdown and amplification efficiencies. All the sequences for transfection are presented in Supplementary Table 1.

### qRT-PCR analysis

TRIzol reagent (Invitrogen) was utilized to extract total RNA from corresponding samples. Transcriptor First Strand cDNA Synthesis Kit (Roche, Penzberg, Germany) was applied for reverse transcription of total RNA based on the instructions. The qRT-PCR of 20 μl volume was conducted through using FastStart Universal SYBR Green Master (Roche) and C1000 Thermal Cycler (Bio-Rad, Hercules, CA). Glyceraldehyde 3-phosphate dehydrogenase (GAPDH) and U6 were used to be internal controls for normalization. The 2^-ΔΔCt^ method was utilized to calculate the relative expression level after normalization. All the primer sequences are listed in Supplementary Table 1.

### Western blot

Radio immunoprecipitation assay (RIPA) lysis buffer (Beyotime, Beijing, China) was utilized to lyse corresponding samples. Bicinchoninic acid (BCA) protein assay kit (Beyotime) was applied to mensurate the protein concentration. 12% sodium dodecyl sulfate-polyacrylamide gel electrophoresis (SDS-PAGE) and 0.45 μm polyvinylidene fluoride (PVDF) membrane (Millipore, Billerica, MA) were used to separate and transfer target protein, respectively. After blocking, diluent primary and secondary antibodies (Abcam, Cambridge, MA) were utilized to incubate the membranes sequentially. BeyoECL kit (Beyotime) was used to visualize the protein bands. GAPDH was applied as an endogenous control.

### Cell viability assays

According to the operational manual, proliferative capacity of CCA cells was detected by using CCK-8 kit (Dojindo, Kumamoto, Japan). Treated cells were seeded into 96-well plates in 100 μl complete culture medium. The samples were supplemented with 10 μl/well CCK-8 reagent prior to each test and then incubated for 2 h. A microplate reader (Tecan, Männedorf, Switzerland) was used to measure 450 nm absorbance every 24 h until 96 h. Afterwards, EdU assay (Ribobio, Guangzhou, China) was also conducted to monitor cellular viability. Transfected cells were cultured with 50 μl EdU diluent for 2 h. The cell samples were fixed in paraformaldehyde and sequentially stained with Apollo 567 working solution and Hoechst 33342 reaction solution in the dark. A fluorescence microscope (Leica, Wetzlar, Germany) was utilized to capture images. Next, colony formation assay was conducted to detect colony-forming ability of CCA cells. Transfected cells were seeded into 6-well plates and cultured in an incubator for 12 days. The cellular colonies were fixed in paraformaldehyde and stained with crystal violet (Beyotime). The colony numbers were counted via visual observation.

### Spheroid formation assay

24-well ultra-low attachment plates (Corning, Corning, NY) were utilized to cultivate CCA cells in serum-free DMEM/F12 medium supplemented with 1× B27, 20 ng/ml epidermal growth factor (EGF), 20 ng/ml basic fibroblast growth factor (bFGF, Invitrogen), 100 U/ml penicillin and 0.1 mg/ml streptomycin (Beyotime). After ten days, spheroid number and size were evaluated under an inverted optical microscope (Leica). The formula for quantification was spheroid formation efficiency = colonies/input cells × 100%).

### Migration and invasion assays

Wound healing assay was performed to evaluate motility of CCA cells. Transfected cells with 80-90% confluence were linearly scratched on the surface and cultured with serum-free medium. Cellular migration was assessed at 0 h and 48 h by gauging wound distance. Next, transwell chambers (Corning) precoated without and with Matrigel (BD, San Jose, CA) were used to test the capability of migration and invasion, respectively. Treated cells were planted into the upper chamber with serum-free medium, and complete culture medium was added into the lower chamber. After culture for 24 h, the upper cells were wholly erased and the remaining cells were stained with crystal violet. An inverted optical microscope (Leica) was used to observe and calculate the colored cells.

### ChIP assay

ChIP assay (Millipore) was performed to verify binding of transcription factor to promoter according to producer’s instructions. The cells were fixed through using formaldehyde to generate cross-linked protein and DNA, and then chromatin fragments were achieved by using sonication. Specific antibody was utilized to generate immunoprecipitations, and IgG (Millipore) was regarded as negative control. The recuperated DNA fragments were evaluated via qRT-PCR. Supplementary Table 1 contained all primer sequences.

### Luciferase reporter assay

Luciferase reporter plasmids (Promega, Madison, WI) were constructed with wild type and mutant type, respectively. Firefly luciferase represented the primary reporter that monitored the binding of protein/miRNA to cloned target sequences. Renilla luciferase was regarded as a control reporter for normalization. The luciferase reporter plasmids and regulating factors were cotransfected into CCA cells through applying Lipofectamine 3000 reagent. After 48 h, luciferase activity was measured via a dual luciferase reporter assay kit (Promega).

### Subcellular fractionation assay

A PARIS kit (Life Technologies, Carlsbad, CA) was used to isolate cytoplasmic and nuclear components according to producer’s instructions. CCA cells were lysed by using cell fractionation buffer and then centrifugated to acquire upper cytoplasmic components. The remaining deposition was disposed by applying cell disruption buffer for obtaining nuclear components. The qRT-PCR was utilized to test extracted RNAs from cytoplasm and nucleus, respectively. GAPDH and U6 were separately used as cytoplasmic control and nuclear control.

### RIP assay

A RIP kit (Millipore) was used to conduct RIP assay according to producer’s instructions. CCA cells were lysed by using RIP lysis buffer. The magnetic beads linked with anti-AGO2 antibody or control IgG (Millipore) were separately applied to generate immunoprecipitations. Proteinase K was utilized to treat the immunoprecipitations, thereby acquiring purified RNA samples. The qRT-PCR was further used to analyse the extracted RNAs.

### Tumor xenograft experiment

Five-week-old female BALB/c nude mice were purchased from Vital River Laboratory Animal Technology Co., Ltd. (Beijing, China) and fed in specific-pathogen-free environment. The body weight of nude mice was approximately 16g. All the animal works were performed in the Animal Experimental Center of Second Affiliated Hospital of Harbin Medical University. The posterior flanks of mice were subcutaneously injected with transfected CCLP-1 cell suspension. The formula 0.5 × length × width^2^ was used to calculate the tumor volumes every 3 days. After 21 days, all mice were euthanized and tumor weights were measured. The choice of euthanasia for mice was dislocation of cervical vertebra. All animal experiments were approved and supervised by the Animal Care and Use Committee of Second Affiliated Hospital of Harbin Medical University (KY2018-354).

### Statistical analysis

GraphPad Prism 6.0 software (GraphPad Software, La Jolla, CA) and SPSS 20.0 software (IBM SPSS, Armonk, NY) were applied. Data were presented as mean ± standard deviation (SD) based on at least three independent experiments. Comparisons between groups were carried out by using Student’s *t*-test, analysis of variance (ANOVA) and Chi-square test. Kaplan-Meier method and log-rank test were used for survival analysis. Pearson correlation was utilized for correlation analysis. Prognostic risk factors were assessed via univariate and multivariate Cox proportional hazards regression model and ROC analysis. *P* value < 0.05 was considered to be a statistically significant difference.

## Supplementary Material

Supplementary Figure 1

Supplementary Table 1
