# Supplementary material for: AR-induced ZEB1-AS1 represents poor prognosis in cholangiocarcinoma and facilitates tumor stemness, proliferation and invasion through mediating miR-133b/HOXB8
Source: Aging (Albany NY). 2020 Jan 24;12(2):1237–55. doi: 10.18632/aging.102680 (PMC7053610; doi:10.18632/aging.102680)
Supplement: Supplementary Figure 1 [file aging-12-102680-s002..pdf]

SUPPLEMENTARY FIGURE

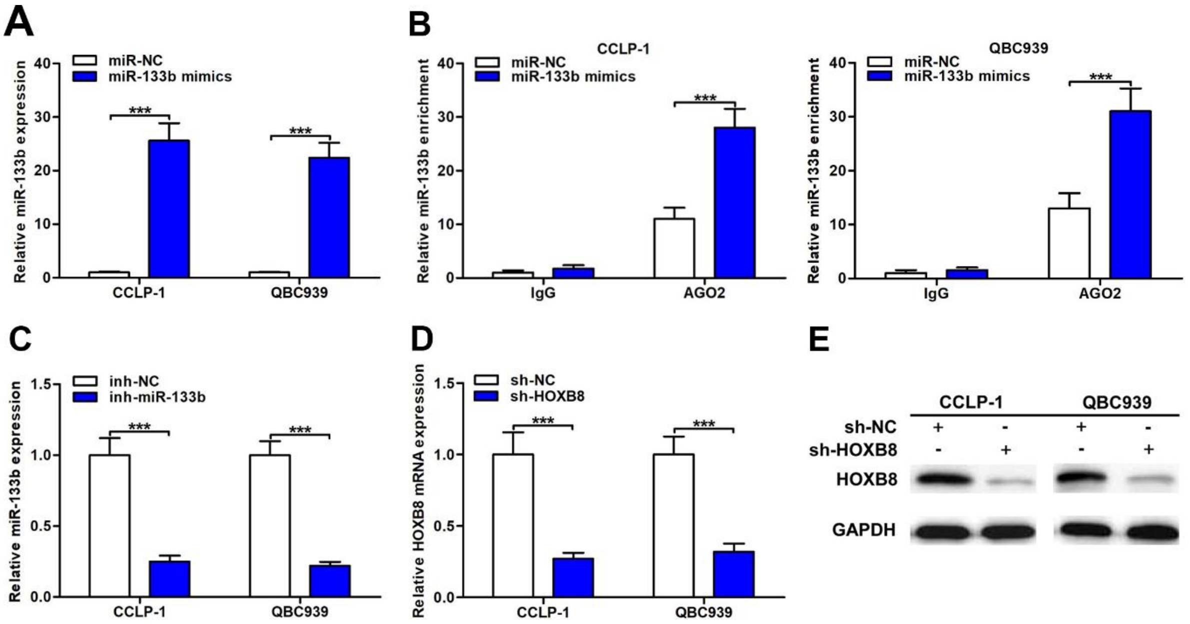

Supplementary Figure 1. Knockdown efficiency and amplification efficiency. (**A**) The amplification efficiency of miR-133b mimics in QBC939 and CCLP-1 cells. (**B**) The enrichment of miR-133b in AGO2 RIP assays. (**C**) The knockdown efficiency of inh-miR-133b in QBC939 and CCLP-1 cells. (**D**, **E**) The knockdown efficiency of sh-HOXB8 in QBC939 and CCLP-1 cells was analysed by qRT-PCR and western blot. $^{***}P <$ 0.001.
