# Supplementary material for: AR-induced ZEB1-AS1 represents poor prognosis in cholangiocarcinoma and facilitates tumor stemness, proliferation and invasion through mediating miR-133b/HOXB8
Source: Aging (Albany NY). 2020 Jan 24;12(2):1237–55. doi: 10.18632/aging.102680 (PMC7053610; doi:10.18632/aging.102680)
Supplement: Supplementary Table 1 [file aging-12-102680-s001..pdf]

SUPPLEMENTARY TABLE

Supplementary Table 1. Primer sequences for qRT-PCR and sequences for siRNA.

Nucleic acids	Sequences
ZEB1-AS1	Forward primer: 5'-TCCCTGCTAAGCTTCCTTCAGTGT-3'
	Reverse primer: 5'-GACAGTGATCACTTTCATATCC-3'
GAPDH	Forward primer: 5'-GGGAGCCAAAAGGGTCAT-3'
	Reverse primer: 5'-GAGTCCTTCCACGATACCAA-3'
ZEB1-AS1 promoter E1 region	Forward primer: 5'-GGGCTTGGATGGCGCT-3'
	Reverse primer: 5'-ACCACATGTTCAGGTCTCGAT-3'
ZEB1-AS1 promoter E2 region	Forward primer: 5'-TTTCTGGTTATCTCGGGGCG-3'
	Reverse primer: 5'-CTCTCGCCACAGGAACTGTC-3'
ZEB1-AS1 promoter E3 region	Forward primer: 5'-TCCTGTCTAGAAGCAGATACGAA-3'
	Reverse primer: 5'-TGCAATAGCCTATGCTCCACT-3'
U6	Forward primer: 5'-GCTTCGGCAGCACATATACTAAAAT-3'
	Reverse primer: 5'-CGCTTCACGAATTTGCGTGTCAT-3'
miR-133a-3p	Forward primer: 5'-ACACTCCAGCTGGGTTTGTCCCCTTCAAC-3'
	Reverse primer: 5'-TGGTGTCGTGGAGTCG-3'
miR-185-5p	Forward primer: 5'-TGAGGAGCCGATCACGTC-3'
	Reverse primer: 5'-GTGCCGGTGCAGAGGT-3'
miR-133b	Forward primer: 5'-CTCAGCTTTGGTCCCCTTCAAC-3'
	Reverse primer: 5'-GTGCAGGGTCCGAGGT-3'
miR-186-5p	Forward primer: 5'-AAGAATTCTCCTTTTGGGCT-3'
	Reverse primer: 5'-GTGCGTGTCGTGGAGTCG-3'
miR-342-3p	Forward primer: 5'-TCCTCGCTCTCACACAGAAATC-3'
	Reverse primer: 5'-TATGGTTGTTCACGACTCCTTCAC-3'
miR-4739	Forward primer: 5'-GCTGGGACATTGAAAGTCTCA-3'
	Reverse primer: 5'-GATGTTCCCATCGGCGTGTC-3'
miR-4306	Forward primer: 5'-AAAGCGCCGCTGGAGAGA-3'
	Reverse primer: 5'-TATGGTTGTTCACGACTCCTTCAC-3'
miR-499a-5p	Forward primer: 5'-ATGTAGCGTGCGACCG-3'
	Reverse primer: 5'-CAGGCTGACGCACTCTGTGCT-3'
miR-5590-3p	Forward primer: 5'-CCCCCTTGTCATGTTCCTGATCTT-3'
	Reverse primer: 5'-GCAGAGGAAATGAAACCAGTATGT-3'
HOXB8	Forward primer: 5'-ACGTGCTTCTTTGTAATGACCA-3'
	Reverse primer: 5'-TGTAACAATTGCCCACAGCG-3'
si-ZEB1-AS1-1	5'-GGGTGTAAAAGAACCCGTA-3'
si-ZEB1-AS1-2	5'-GAATCATAACCTTTATTGCA-3'
sh-HOXB8	5'-GCTCTTATTTCGTCAACTCACTGTTCTCC-3'
